# CDMO: Chaotic Dwarf Mongoose Optimization Algorithm for feature selection

**DOI:** 10.1038/s41598-023-50959-8

**Published:** 2024-01-06

**Authors:** Mohammed Abdelrazek, Mohamed Abd Elaziz, A. H. El-Baz

**Affiliations:** 1 Department of Mathematics, Faculty of Science, Damietta University, New Damietta, 34517 Egypt; 2 Department of Mathematics, Faculty of Science, Zagazig University, Zagazig, 44519 Egypt; 3 Artificial Intelligence Research Center (AIRC), Ajman University, Ajman 346, UAE; 4 MEU Research Unit, Middle East University, Amman, 11831 Jordan; 5 Department of Electrical and Computer Engineering, Lebanese American University, Byblos, 13-5053 Lebanon; 6 Department of Computer Science, Faculty of Computers and Artificial Intelligence, Damietta University, New Damietta, 34517 Egypt

**Keywords:** Computer science, Software

## Abstract

In this paper, a modified version of Dwarf Mongoose Optimization Algorithm (DMO) for feature selection is proposed. DMO is a novel technique of the swarm intelligence algorithms which mimic the foraging behavior of the Dwarf Mongoose. The developed method, named Chaotic DMO (CDMO), is considered a wrapper-based model which selects optimal features that give higher classification accuracy. To speed up the convergence and increase the effectiveness of DMO, ten chaotic maps were used to modify the key elements of Dwarf Mongoose movement during the optimization process. To evaluate the efficiency of the CDMO, ten different UCI datasets are used and compared against the original DMO and other well-known Meta-heuristic techniques, namely Ant Colony optimization (ACO), Whale optimization algorithm (WOA), Artificial rabbit optimization (ARO), Harris hawk optimization (HHO), Equilibrium optimizer (EO), Ring theory based harmony search (RTHS), Random switching serial gray-whale optimizer (RSGW), Salp swarm algorithm based on particle swarm optimization (SSAPSO), Binary genetic algorithm (BGA), Adaptive switching gray-whale optimizer (ASGW) and Particle Swarm optimization (PSO). The experimental results show that the CDMO gives higher performance than the other methods used in feature selection. High value of accuracy (91.9–100%), sensitivity (77.6–100%), precision (91.8–96.08%), specificity (91.6–100%) and F-Score (90–100%) for all ten UCI datasets are obtained. In addition, the proposed method is further assessed against CEC’2022 benchmarks functions.

## Introduction

Feature selection is one of the major steps in pattern recognition and classification since it aims to eliminate the redundant and irrelevant features within a dataset. It can be challenging to decide which features are useful without prior knowledge. As a result, numerous feature selection techniques are used to select the best features which give superior performance^1^. Particularly in applications, each dataset contains numerous significant numbers of features. The key objective of feature selection is to have a greater understanding of the methodology that produced the data in order to identify a subset of pertinent features from the vast pool of available features^2^.

There are two main types of feature selection techniques. First, filtering techniques that don't rely on learning algorithms but rather specific data attributes. In contrast, wrapper approaches evaluate the chosen subset of features using learning algorithms. Although wrapper methods are computationally expensive, they are more accurate than filter approaches^3^. In general, feature selection is typically a multi-objective optimization problem. Its two main goals are to reduce the feature space and gives high performance. When there is a tradeoff between these two objectives, which they frequently do, the best choice must be made^4^.

Recently, meta-heuristic optimization algorithms are frequently used for finding the most discriminative features. The most methods that have been studied are Particle Swarm Optimization (PSO)^5^, Ant Colony Optimization (ACO)^6^, Genetic Algorithm (GA)^7^, Genetic Programming (GP)^8^, Simulated Annealing (SA)^9^, Differential Evolution (DE)^10^, Cuckoo Search (CS)^11^, Artificial Immune Systems Algorithm (AIS)^12^, Tabu Search (TS)^13^, and Whale Optimization algorithm (WOA)^14^. In other hand, there are studies including multi objective and its hybrid versions that have been published with these classical meta-heuristic algorithms. The theorem of No-Free-Launch (NFL) is the reason of studies multiplicity where no algorithm can give best solution for all problems, so there is always a probability to find better solution with new meta-heuristic algorithm, that’s why there are hundreds of studies in this field^15^.

Xue et al.^16^ provided first multi-objective method for feature selection using PSO algorithm, the experiments on 12 Benchmark dataset showed better results for their method comparing traditional one. Emary et al.^17^ used Anti Lion Optimization (ALO) in two approaches and compared the results with other common algorithms such GA and Big Bang algorithm (BBA) which proved the capability of their proposed method to find optimal features using 20 UCI dataset. Also, he employed Lèvy flight random walk with Ant Lion Optimizer (ALO) and the results showed its improvement comparing to the native ALO using 21 Benchmark dataset^3^. Genetic algorithms were the earlier method that have been used in feature selection, Aalaei et al.^18^ developed feature selection method by genetic algorithm (GA) to diagnose breast cancer using Wisconsin breast cancer dataset. Their experiments improved the accuracy, specificity and sensitivity. Ferriyan et al.^19^ used GA on NSL-KDD Cup 99 datasets. By using one point crossover instead of two, they get better results on the datasets they used comparing to original method.

The artificial bee colony (ABC)^20^ algorithm is a simple, flexible, and efficient meta-heuristic optimization algorithm. However, it can suffer from slow convergence due to its lack of a powerful local search capability. Etminaniesfahani et al.^21^ overcome this weakness by hybridizing the ABC algorithm with Fibonacci indicator algorithm (FIA)^22^, calling the new algorithm by ABFIA^21^. Their hybrid algorithm combines the strengths of the artificial bee colony (ABC) algorithm and the Fibonacci indicator algorithm (FIA) by combines the global exploration of the FIA with the local exploitation of the ABC. They demonstrate that the hybrid algorithm outperforms the ABC and FIA algorithms and produces superior results for a variety of optimization functions that are commonly used in the literature, including 20 scalable basic and 10 complex CEC2019 test functions. Akinola et al.^23^ combined the binary dwarf mongoose BDMO algorithm with simulated annealing (SA) algorithm and compared it with other 10 algorithms. The results showed that their proposed (BDMSAO) method is better than other algorithms.

Eluri et al.^24^ introduces a novel wrapper-based method called BGEO-TVFL for addressing feature selection challenges. Their proposed BGEO-TVFL method employs Binary Golden Eagle Optimizer with Time Varying Flight Length (TVFL) to enhance feature selection. Their method adapts the Golden Eagle Optimizer (GEO), a swarm-based meta-heuristics algorithm, for discrete feature selection. Their work explores various transfer functions and incorporates TVFL for a balanced exploration–exploitation trade-off in GEO. They measure their performance evaluation by using UC Irvine datasets and comparison with standard feature selection approaches namely BAT, ACO, PSO, GWO, GA, CS, IG, CFS, GR. The obtained results reveal the superiority of BGEO-TVFL. Their method is tested using CEC benchmark functions, demonstrating its effectiveness in addressing dimensionality reduction issues compared to existing methods.

Chaotic Binary Pelican Optimization Algorithm is proposed by Eluri and Devarakonda^25^, their proposed algorithm leverages the principles of chaos theory in a binary context to enhance the efficiency of the Pelican Optimization Algorithm for this purpose. In this binary variant, they introduce chaos to improve exploration and exploitation capabilities. Their algorithm aims to address the challenges of feature selection, particularly in handling large datasets and optimizing performance. Their proposed Chaotic Binary Pelican Optimization Algorithm is presented as a promising solution for improving feature selection outcomes in data analysis tasks.

Feature Selection with a Binary Flamingo Search Algorithm and a Genetic Algorithm is discussed by Eluri and Devarakonda^26^. They evaluate the performance of HBFS-GA using 18 different UCI datasets and various metrics. The results demonstrate that HBFS-GA outperforms existing wrapper-based and filter-based FS methods.

In the new proposed technique for feature selection, the DMO algorithm is used with chaotic maps to select the best prominent features. The DMO is used to explore and find minimal possible features in the datasets. The K-Nearest Neighbor (KNN) is used to evaluate the performance of the selected features. The results obtained by the proposed method proved their efficiency and gave better performance over other related state-of-the-art methods. We can summarize the main contribution of this paper as follows:

- Propose a new hybrid feature selection method called CDMO based on improving the performance of DMO using chaotic maps.
- Evaluate the proposed CDMO method using ten UCI datasets employing the K-nearest Neighbors (KNN) as a classifier to prove its effectiveness.
- The results obtained by the proposed CDMO give superior performance than the original DMO algorithm and with other well-known meta-heuristic-based feature selection methods.
- On the CEC’22 test suite, the effectiveness and solution quality generated by our proposed method are computed and compared by all 9 chaotic maps and compared with state-of-the-art algorithms.

The rest part of this study is organized as follows: Section "Background" presents background on DMO algorithm and chaotic maps. Section "The proposed CDMO for feature selection" explains the proposed model. Experimental results and analysis are discussed in Section "Experimental results". Finally, the conclusion is summarized in Section "Conclusion and future work".

## Background

### Dwarf Mongoose Optimization Algorithm (DMO)

DMO^27﻿^ is a meta-heuristic method that simulates the foraging behavior of the dwarf mongoose that uses its compensatory behavioral adaptations. The mongoose has two main compensatory behavioral adaptations, which are:

1. Prey size, group size, and space utilization.
2. Food Provisioning.

Large prey items, which could provide food for the whole group, are not amenable to capture by dwarf mongooses. Due to the lack of a killing bite and organized pack hunting, the dwarf mongoose has evolved a social structure that allows each individual to survive independently and move from one location to another. The dwarf mongoose lives a semi-nomadic lifestyle in an area big enough to accommodate the entire colony. Because no previously visited sleeping mound is returned, the nomadic lifestyle ensures that the entire territory is explored and prevents over-exploitation of any one area^27^.

#### Population initialization

The candidate populations of the mongooses (X) are initialized using Eq. (1). Between the upper bound (UB) and lower bound (LB) of the given problem, the population is generated stochastically.

1 $$X=\left[\begin{array}{ccccc}{x}_{\mathrm{1,1}}& {x}_{\mathrm{1,2}}& ...& {x}_{1,d-1}& {x}_{1,d}\\ {x}_{\mathrm{2,1}}& {x}_{\mathrm{2,2}}& ...& {x}_{2,d-1}& {x}_{2,d}\\ & \vdots & {x}_{i,j}& \vdots & \\ {x}_{n,1}& {x}_{n,2}& ...& {x}_{n,d-1}& {x}_{n,d}\end{array}\right]$$

where $X$ is the populations, created at random by Eq. (2), ${x}_{i,j}$ stands for the location of the *j*th dimension in the *i*th population, *n* stands for population size, and *d* stands for the problem dimension.

2 $${x}_{i,j}=VarMin+rand\times \left(VarMax- VarMin\right)$$

where *rand* is a random number between [0, 1], *VarMax* and *VarMin* are upper and lower bound of the problem. The best solution over iteration is the best-obtained solution so far.

The fitness of each solution is calculated after the population has been initiated. Equation (3) calculates the probability value for each population fitness, and the alpha female (α) is chosen based on this probability.

3 $$\alpha =\frac{fi{t}_{i}}{{\sum }_{i=1}^{n}fi{t}_{i}}$$

The n-*bs* is equal to the number of mongooses in the alpha group. Where *bs* represents the number of nannies. *Peep* is the alpha female's vocalization that directs the family's path.

The DMO applies the formula from Eq. (4) to provide a candidate food position.

4 $${X}_{i+1}={X}_{i}+phi*peep$$

where *phi* is a uniformly distributed random number [− 1,1], after each iteration, the sleeping mound is specified as in Eq. (5).

5 $$s{m}_{i}=\frac{fi{t}_{i+1}-fi{t}_{i}}{\mathit{max}\{|fi{t}_{i+1},fi{t}_{i}|\}}$$

The average value of the sleeping mound found is given by Eq. (6).

6 $$\varphi =\frac{{\sum }_{i=1}^{n}s{m}_{i}}{n}$$

The mongooses are known to avoid returning to the previous sleeping mound, so the scouts search for the next one to ensure exploration. The scout mongoose is simulated by Eq. (7).

7 $${X}_{i+1}=\left\{\begin{array}{c}{X}_{i}-CF*phi*rand*\left[{X}_{i}-\overrightarrow{M}\right] if {\varphi }_{i+1}>{\varphi }_{i}\\ {X}_{i}+CF*phi*rand*\left[{X}_{i}-\overrightarrow{M}\right] elsewhere\end{array}\right.$$

where, $CF=(1-\frac{iter}{Ma{x}_{iter}}{)}^{\left(2\frac{iter}{Ma{x}_{iter}}\right)}$ indicates the variable, which decreases linearly with each iteration, that controls the group's collective-volatile movement. $\overrightarrow{M}={\sum }_{i=1}^{n}\frac{{x}_{i}\times s{m}_{i}}{{X}_{i}}$ is the vector that controls the mongoose's movement to its new sleeping mound.

### Chaotic maps

**Figure 1:**
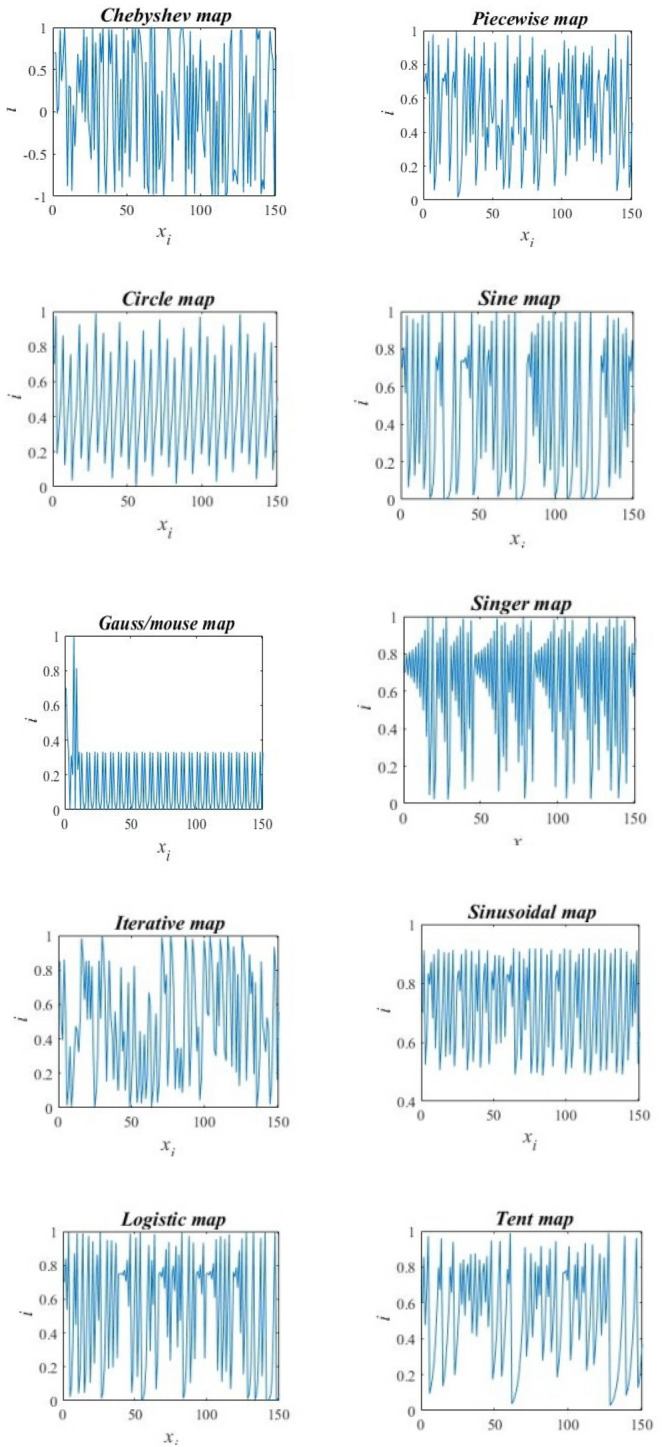
Ten chaotic maps.

Chaos is a phenomenon that can exhibit non-linear changes in future behavior when its initial condition is even slightly altered. Additionally, it is described as a semi-random behavior generated by nonlinear deterministic systems^28^. One of main search algorithms is Chaos Optimization Algorithm (COA) which moves variables and parameters from the chaos to the solution space. It relies on determining the global optimum for stochastic, regular, and periodicity chaotic motion properties. Due to its simplicity and speedily convergence, COA has widely used in last ten years in many papers e.g.,^29–32^. To obtain the chaotic sets, we have used ten well known one-dimensional maps that have been used frequently in literature. Figure 1 shows that the maps have different behaviors which allow testing the behavior of DMO on different maps.

## The proposed CDMO for feature selection

**Figure 2:**
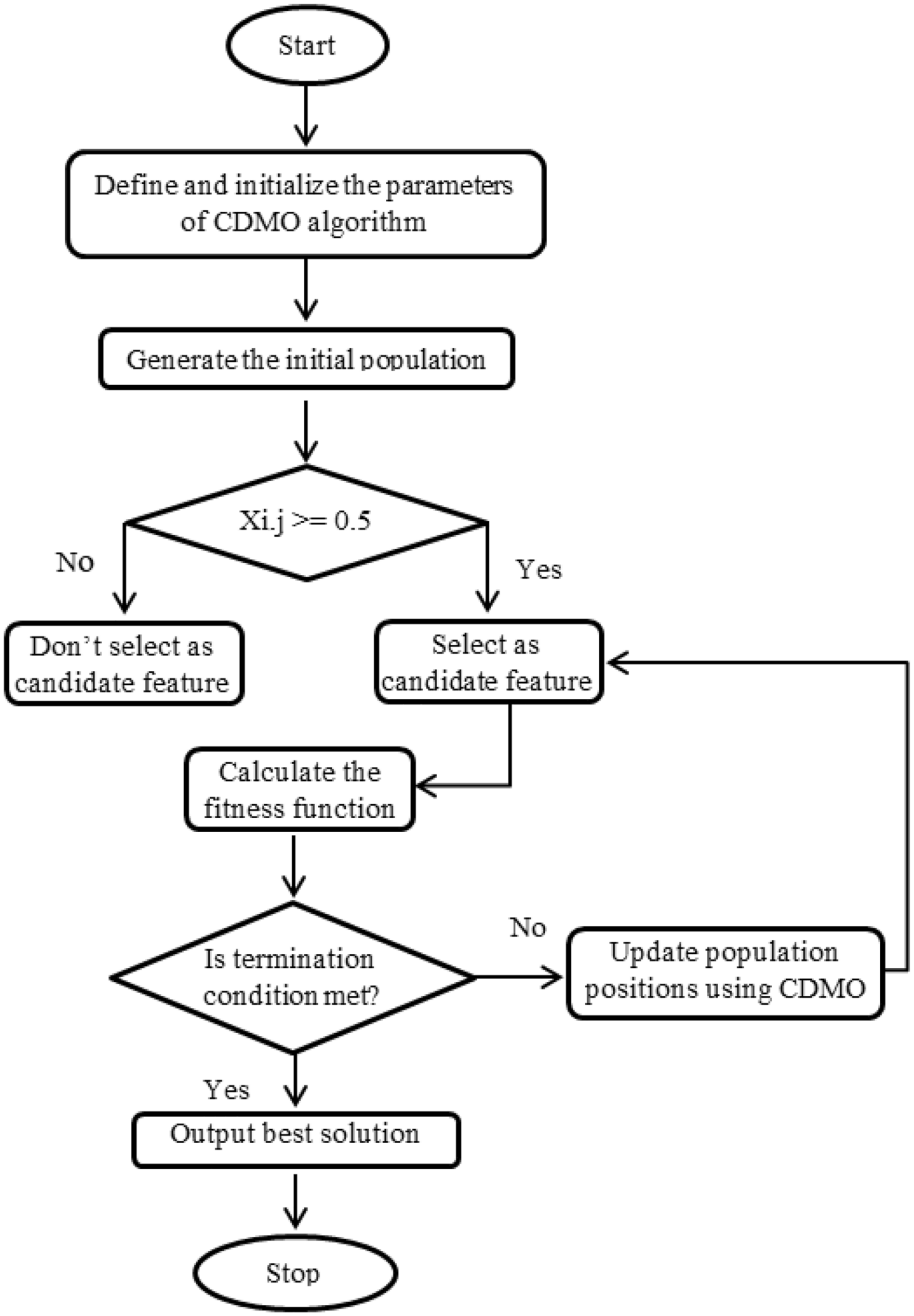
Flowchart of the proposed CDMO algorithm.

In this study, an alternative feature selection technique is proposed using the Chaotic Dwarf Mongoose Optimization (CDMO) as in Fig. 2. Random numbers which are used in Eq. (7) are replaced by chaotic maps to avoid returning to same sleeping mound.

8 $${X}_{i+1}=\left\{\begin{array}{ll}{X}_{i}-CF*phi*\rho *\left[{X}_{i}-\overrightarrow{M}\right] &\quad if {\varphi }_{i+1}>{\varphi }_{i}\\ {X}_{i}+CF*phi*\rho *\left[{X}_{i}-\overrightarrow{M}\right] &\quad else\end{array}\right.$$

where $\rho$ is value obtained from well-known chaotic maps which reported in Table 1.

**Table 1:** Ten chaotic maps.

#Map	Name	Definition	Range
1	Chebyshev	${P}_{q+1}={\text{cos}}(q{cos}^{-1}({P}_{q}))$	(-1,1)
2	Circle	${P}_{q+1}=mod\left({P}_{q}+r-\left(\frac{l}{2\pi }\right){\text{sin}}2\pi {P}_{q}\right),1),l=0.5 and r=0.2$	(0,1)
3	Gauss/mouse	$${P}_{q+1}=f\left(x\right)=\left\{\begin{array}{c}1 , {p}_{q}=0\\ \frac{1}{mod({p}_{q},1)}, otherwise\end{array}\right.$$	(0,1)
4	Iterative	${P}_{q+1}={\text{sin}}\left(\frac{l\pi }{{P}_{q}}\right),l=0.7$	(-1,1)
5	Logistic	${P}_{q+1}=l{P}_{q}(1-{P}_{q}),l=4$	(0,1)
6	Piecewise	$${P}_{q+1}=f\left(x\right)=\left\{\begin{array}{c}\frac{{P}_{q}}{l}, 0\le {P}_{q}<1\\ \frac{{P}_{q}-l}{0.5-l}, 1\le {P}_{q}<0.5\\ \begin{array}{cc}\frac{1-l-{P}_{q}}{0.5-l},& 0.5\le {P}_{q}<1-l\end{array}\\ \begin{array}{cc}\frac{1-{P}_{q}}{l},& 1-l\le {P}_{q}<1\end{array}\end{array},l=0.4\right.$$	(0,1)
7	Sine	${P}_{q+1}=\frac{l}{4}{\text{sin}}(\pi {P}_{q}),l=4$	(0,1)
8	Singer	${P}_{q+1}=\mu \left(7.86{P}_{q}-23.31{P}_{q}^{2}+28.75{P}_{q}^{3}-13.302875{P}_{q}^{4}\right),\mu =1.07$	(0,1)
9	Sinusoidal	${P}_{q+1}=l{P}_{q}^{2}{\text{sin}}(\pi {P}_{q}),l=2.3$	(0,1)
10	Tent	$${P}_{q+1}=\left\{\begin{array}{c}\frac{{P}_{q}}{0.7}, {P}_{q}<0.7\\ \frac{10}{3}(1-{P}_{q}), {P}_{q}\ge 0.7\end{array}\right.$$	(0,1)

After that, we have set the dimension of the problem, which is *d* in Eq. (1) as the number of features then give value of $VarMin$ and $VarMax$ in Eq. (2) as 0 and 1, respectively. For each row in Eq. (1) (i.e., the position of each element in ${X}_{i}$) is threshold by 0.5, since the values are set between 0 and 1. After that, elements with positions > 0.5 are considered as candidate features, while elements with positions < 0.5 are not considered in this solution.

9 $${X}_{i,j}=\left\{\begin{array}{ll}1&\quad { x}_{i,j}>0.5\\ 0 &\quad Otherwise\end{array}\right.$$

The candidate features are then applied to the fitness function which calculates the classification accuracy of *k*-nearest neighbor classifier using the applied candidate features.

10 $$Fitness= \frac{Number\,of\,wrong\,classified }{Total\,numbers\,of\,instances}+\frac{|{X}_{i}|}{d}$$

Each time the fitness function is invoked the dataset is divided using the holdout method to 80% training dataset and 20% testing dataset. Algorithm 1 and Fig. 2 show the algorithm and the flowchart of the proposed technique, respectively.

**Figure:**
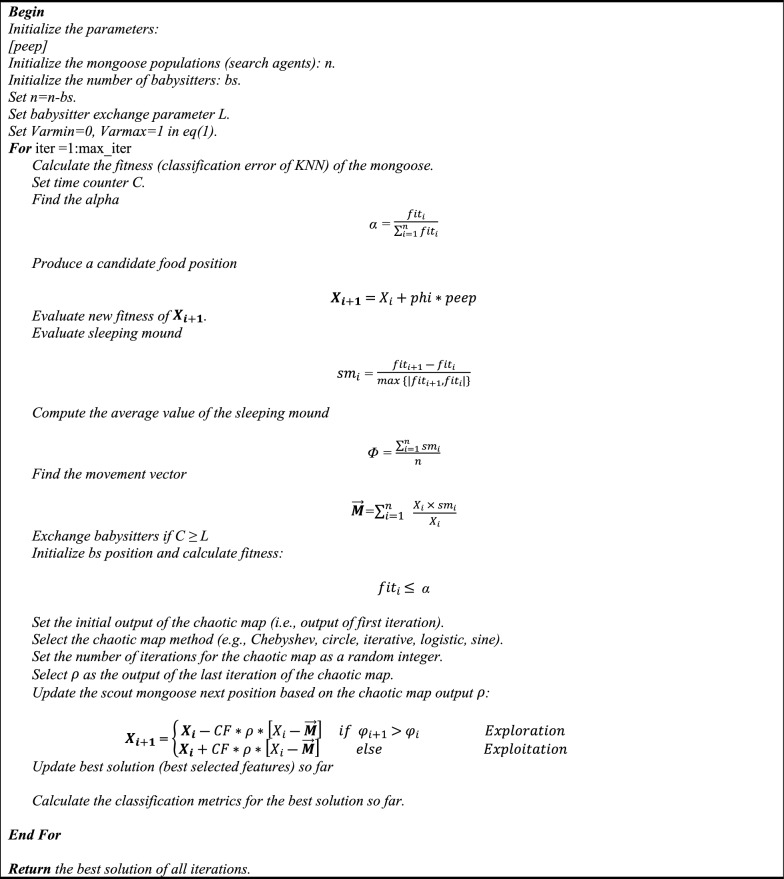
**Algorithm 1** Steps of the developed method.

## Experimental results

### Dataset and parameters setting

Table 2 lists the 10 datasets that were used in this study which are come from the well-known UCI data warehouse^33^. They have been chosen with different dimensions and different patterns to evaluate the performance of the proposed method on several complexities.

**Table 2:** Datasets used in this study.

Index	Dataset	# dims	# instances
1	base_BreastEW	30	569
2	base_Exactly	13	1000
3	base_M-of-n3	13	1000
4	breastEW	30	569
5	CongressEW	16	435
6	Ionosphere	34	351
7	KrvskpEW	36	3196
8	SonarEW	60	208
9	SpectEW	22	267
10	WaveformEW	40	5000

*K*-nearest neighbor (*K*NN) is employed as a classifier in this study as it is one of the most common and simplest learning algorithms, it is trained using the training dataset, then, tested using the testing part, which ensures higher reliability. To simplify the evaluation process, we choose *K* = 5 in *K*NN as 5NN^34^.

### Performance metrics

In this study we have used two types of metrics to evaluate the performance which are Fitness metrics and classification Metrics.

In fitness metrics we have used four statistical measurements which are the worst, best, mean fitness value and the standard deviation which are mathematically defined as following

11 $${\text{BestFitness}}= {{\text{Max}}}_{{\text{i}}=1}^{{N}_{r}}{{\text{BS}}}_{{\text{i}}},$$

12 $$\mathrm{Worst Fitness}= {\mathit{Min}}_{i=1}^{{N}_{r}}{{\text{BS}}}_{{\text{i}}},$$

13 $$\mathrm{Mean Fitness}= \frac{1}{{N}_{r}}\sum_{{\text{i}}=1}^{{N}_{r}}{{\text{BS}}}_{{\text{i}}},$$

14 $$\mathrm{Standard Deviation }\left({\text{SD}}\right)= \sqrt{\frac{\sum_{{\text{i}}=1}^{{N}_{r}}{({{\text{BS}}}_{{\text{i}}}-\upmu )}^{2}}{{N}_{r}}}$$

where *BS* is the best score gained in each iteration and Nr is the number of runs^35^.

The second evaluation was used to evaluate the selected features using classification measures. These measures are accuracy, precision, sensitivity, specificity, and F-Score. Accuracy is a common technique of evaluation, which is defined as the ratio of correctly classified samples to all samples. It’s mathematically defined as following

15 $$Accuracy= \frac{TP+TN}{TP+TN+FP+FN} ,$$

Precision, specificity and sensitivity are proper metrics to measure the performance of classification across unbalanced datasets. While they are not affected by differences in data distribution, therefore these measures are useful for evaluating classification performance in unbalanced learning scenarios^36﻿^. The F-Score metric make combination between precision and sensitivity and it is given by Eq. (19). Therefore, F-Score is suitable in unbalanced scenarios than the accuracy metric. Precision, sensitivity, specificity and F-score measures are defined by the following equations:

16 $$Precision = \frac{TP}{TP+FP}$$

17 $$Sensitivity= \frac{TP}{TP+FN}$$

18 $$Specificity = \frac{TN}{TN+FP}$$

19 $$F-score= \frac{2*\left(precision *Sensitivity\right)}{Precision +Sensitivity}$$

where *TP* is the true positive, *FP* is the false positive, *FN* is the false negative and *TN* represents the true negative.

### Performance of DMO based on ten chaotic maps

To evaluate the performance of the proposed CDMO, 10 different datasets from UCI repository are used. The obtained results are compared with the DMO and other well-known meta-heuristic algorithms namely, PSO^5^, ACO^6^, ARO^37^, HHO^38^, EO^39^, RTHS^40^, RSGW^41^, SSAPSO^42^, BGA^43^ and WOA^14^ algorithms. Each one of them has been performed 25 runs in the same PC specifications. To test the convergence capability, the average 25 runs has been computed and compared for each algorithm. Table 3 illustrates the parameter settings of the algorithms used in this study. The experiments are divided into two sections, the first one is to evaluate the performance of the ten chaotic maps on DMO algorithm as shown in Tables 4 and 5, the second experiments are to compare the best chaotic maps with the six meta-heuristic algorithms DMO, ACO, PSO, ARO, HHO, and WOA as shown in Tables 6 and 7.

**Table 3:** Parameter setting.

Parameter	Value
k-value of KNN	5
Number of populations	20
Number of iterations	100
Problem dimensions	Number of features in the used dataset
Data search domain	[0 1]
Repetition of runs	25
No of babysitters in DMO	3
No of peep in DMO	2
α	1
τ	1
β	0.1
Pheromone in PSO	0.2
B constant in WOA	1
Initial value of chaotic	0.7
Iteration number in chaotic	500

**Table 4:** Accuracy comparison between ten CDMO.

	Accuracy									
	CDMO1	CDMO2	CDMO3	CDMO4	CDMO5	CDMO6	CDMO7	CDMO8	CDMO9	CDMO10
base_exactly	**1**	**1**	**1**	**1**	**1**	**1**	**1**	**1**	**1**	**1**
base_BreastEW	**1**	0.9911	0.9911	0.9823	0.9734	0.9734	0.9911	0.9911	0.9823	**1**
base_M-of-n3	**1**	**1**	**1**	**1**	**1**	**1**	**1**	**1**	**1**	**1**
breastEW	0.9911	0.9823	0.9911	0.9911	0.9911	0.9823	0.9906	**0.9921**	0.9911	0.9646
KrvskpEW	**0.9921**	0.9874	0.9906	0.9859	0.9874	0.9859	**1**	0.9890	0.9843	0.9843
SonarEW	**1**	**1**	0.9268	**1**	**1**	0.9756	0.9056	**1**	**1**	0.9756
SpectEW	0.9622	0.9622	0.9622	0.9056	0.9622	0.8867	0.9065	**0.9722**	0.9433	0.9622
Waveform	0.9042	0.9077	0.8898	0.9058	0.9116	0.8993	0.9885	**0.9192**	0.9016	0.9109
CongressEW	0.9770	0.9655	0.9655	**1**	0.9885	0.9770	0.9885	**1**	0.9885	0.9885
Ionosphere	0.9714	0.9714	0.9428	0.9428	0.9557	0.9285	**0.9871**	0.9571	0.9428	0.9428

Significant values are in bold.

**Table 5:** Average fitness comparison between ten CDMO.

	Average									
	CDMO1	CDMO2	CDMO3	CDMO4	CDMO5	CDMO6	CDMO7	CDMO8	CDMO9	CDMO10
base_exactly	0.0245	0.0264	0.0204	0.0129	0.0197	0.0331	0.0096	**0.0055**	0.0074	0.0234
base_BreastEW	**0.0072**	0.0112	0.0103	0.0215	0.0322	0.0334	0.0138	0.0095	0.0186	**0.0027**
base_M-of-n3	0.0085	0.0085	0.0036	**0.0012**	0.0063	**0.0012**	0.0016	0.0016	0.0037	0.0071
breastEW	0.0123	0.0230	0.0105	0.0184	0.0107	0.0201	0.0157	**0.0101**	0.0160	0.0392
KrvskpEW	0.0137	0.0193	0.0128	0.0168	0.0190	0.0175	0.02	**0.0131**	0.0182	0.0219
SonarEW	0.0136	0.0139	0.1026	0.0065	0.0097	0.0441	0.0988	**0.0058**	0.0173	0.0473
SpectEW	0.0567	0.0490	**0.0379**	0.0958	0.0437	0.1137	0.0989	0.0588	0.0622	0.0511
Waveform	0.1016	0.0966	0.1139	0.0991	0.1004	0.1093	**0.0122**	0.0925	0.1012	0.0944
CongressEW	0.0248	0.0386	0.0345	0.0043	0.0140	0.0247	0.0122	**0.0010**	0.0167	0.0114
Ionosphere	0.0448	0.0307	0.0642	0.0747	**0.0297**	0.0927	0.0481	0.0612	0.0708	0.0667

Significant values are in bold.

**Table 6:** Comparison between CDMO8 and 6 meta-heuristic algorithms in classification metrics.

	Accuracy							Precision						
	ACO	PSO	WOA	DMO	ARO	HHO	CDMO8	ACO	PSO	WOA	DMO	ARO	HHO	CDMO8
base_exactly	0.9796	0.9126	0.8372	**1**	0.98	0.745	**1**	0.9811	0.9266	0.8559	**1**	0.9212	0.9259	**1**
base_BreastEW	0.9823	0.9869	0.9823	0.9876	0.9883	0.97345	**0.9911**	0.9823	0.9869	0.9823	0.9876	0.9838	0.9852	**0.9911**
base_M-of-n3	0.9952	0.9748	0.9691	**1**	1	0.985	**1**	0.9952	0.9748	0.9691	**1**	0.9797	0.9809	**1**
BreastEW	0.9855	0.9837	0.9767	0.9911	0.9883	0.95575	**0.9921**	0.9850	0.9875	0.9771	**0.9912**	0.9832	0.9848	0.9861
KrvskpEW	0.9748	0.9744	0.9721	**0.9894**	0.9874	0.97026	0.9890	0.9777	0.9693	0.9744	0.9910	0.9738	0.9771	**0.9939**
SonarEW	0.9375	0.9619	0.9247	0.9824	0.9834	0.87804	**1**	0.9385	0.9679	0.9259	0.9855	0.9441	0.9559	**1**
SpectEW	0.9003	0.9116	0.8883	0.9198	0.9.32	0.94339	**0.9722**	0.9269	0.9336	0.9199	0.8373	0.9268	0.9044	**1**
Waveform	0.8562	0.8637	0.8556	0.9069	0.8227	0.802	**0.9192**	0.8812	0.8881	0.8810	0.9107	0.8834	0.8908	**0.9188**
CongressEW	0.9711	0.9766	0.9731	0.9880	09,839	0.95402	**1**	0.9524	0.9584	0.9584	0.9925	0.9564	0.9664	**1**
Ionosphere	0.9554	0.9308	0.9354	0.9434	**0.96**	0.92	0.9571	0.9511	0.9171	0.9203	0.9272	0.9295	0.9235	**0.9565**

	Sensitivity							Specificity						
	ACO	PSO	WOA	DMO	ARO	HHO	CDMO8	ACO	PSO	WOA	DMO	ARO	HHO	CDMO8
base_exactly	0.9901	0.9529	0.9282	**1**	0.9678	0.9622	**1**	0.9566	0.8233	0.6374	**1**	0.9094	0.8964	**1**
base_BreastEW	0.9702	0.9761	0.9702	**0.9943**	0.9777	0.9796	0.9859	0.9894	0.9932	0.9894	0.9761	0.9794	0.9873	**1**
base_M-of-n3	0.9939	0.9630	0.9506	**1**	0.9768	0.9726	**1**	0.9958	0.9816	0.9799	**1**	0.995	0.9908	**1**
BreastEW	0.9761	0.9685	0.9603	0.9947	0.9749	0.9746	**1**	0.9911	**0.9926**	0.9865	0.9837	0.9844	0.9894	0.9761
KrvskpEW	0.9697	0.9777	0.9672	**0.9887**	0.9758	0.9774	0.9849	0.9796	0.9714	0.9766	0.9901	0.9867	0.9792	**0.9934**
SonarEW	0.9472	0.9618	0.9375	0.9768	0.9558	0.9580	**1**	0.9263	0.9621	0.9100	0.9872	0.9679	0.9521	**1**
SpectEW	0.9504	**0.9580**	0.9428	0.7765	0.9069	0.8961	0.8181	0.7090	0.7345	0.68	0.9573	0.888	0.7772	**1**
Waveform	0.9045	0.9087	0.9039	0.8991	0.9040	0.9039	**0.9218**	0.7618	0.7758	0.7612	0.9145	0.8762	0.8046	**0.9166**
CongressEW	0.9781	0.9831	0.9738	0.9880	0.9807	0.9814	**1**	0.9726	0.9726	0.9727	0.9881	0.9843	0.9765	**1**
Ionosphere	0.9822	0.9831	0.9866	**0.9911**	0.9857	0.9866	0.9777	0.9072	0.8368	0.8432	0.8576	0.854	0.8660	**0.92**

	F-measure													
	ACO	PSO	WOA	DMO	ARO	HHO	CDMO8							
base_exactly	0.9854	0.9390	0.8890	**1**	0.9845	0.95783	**1**							
base_BreastEW	0.9760	0.9822	0.9760	0.9901	0.9993	0.98847	**0.9929**							
base_M-of-n3	0.9934	0.9652	0.9576	**1**	**1**	0.98587	**1**							
BreastEW	0.9804	0.9778	0.9684	0.9929	0.9865	0.98260	**0.9930**							
KrvskpEW	0.9736	0.9734	0.9707	**0.9898**	0.9838	0.98143	0.9894							
SonarEW	0.9419	0.9645	0.9303	0.9808	0.9782	0.96310	**1**							
SpectEW	0.9380	0.9451	0.9304	0.7963	**0.9455**	0.89073	0.90							
Waveform	0.8927	0.8982	0.8922	0.9047	0.8952	0.89737	**0.9203**							
CongressEW	0.9680	0.9700	0.9655	0.9902	0.9830	0.97957	**1**							
Ionosphere	0.9660	0.9485	0.9517	0.9577	**0.9698**	0.95973	0.9670							

Significant values are in bold.

**Table 7:** Comparison between CDMO8 and 6 meta-heuristic algorithms in fitness metrics.

	Average							Best						
	ACO	PSO	WOA	DMO	ARO	HHO	CDMO8	ACO	PSO	WOA	DMO	ARO	HHO	CDMO8
base_exactly	0.0811	0.0995	0.1738	0.0125	0.0214	0.1181	**0.0055**	0.0204	0.0874	0.1627	**0.0125**	0.002	0.0708	0.0264
base_BreastEW	0.0202	0.0141	0.0181	0.0153	0.0149	0.0174	**0.0095**	0.0176	0.0116	0.0176	0.0123	0.0116	0.0148	**0.0088**
base_M-of-n3	0.0200	0.0280	0.0365	0.0085	0.0025	0.0281	**0.0016**	0.0048	0.0252	0.0308	0.0085	**0**	0.0173	0.0016
BreastEW	0.0168	0.0178	0.0244	0.0127	0.0151	0.0196	**0.0101**	0.0144	0.0162	0.0232	**0.0088**	0.0116	0.0157	**0.0088**
KrvskpEW	0.0274	0.0268	0.0286	0.0165	0.0187	0.0276	**0.0131**	0.0251	0.0255	0.0278	**0.0105**	0.0125	0.0222	0.0109
SonarEW	0.0735	0.0457	0.0778	0.0371	0.0309	0.0656	**0.0058**	0.0624	0.0351	0.0752	0.0175	0.0165	0.0476	**0.0139**
SpectEW	0.1076	0.0891	0.1133	0.0881	0.0938	0.1033	**0.0588**	0.0989	0.0853	0.1117	0.0802	0.0867	0.094	**0.0377**
Waveform	0.1453	0.1392	0.1458	0.0997	0.1870	0.1434	**0.0925**	0.1438	0.1361	0.1444	0.0930	0.1772	0.1293	**0.0807**
CongressEW	0.0997	0.0997	0.0278	0.0146	0.0179	0.0757	**0.0010**	0.0226	0.0226	0.0268	0.0120	0.0160	0.021	**0.0010**
Ionosphere	**0.0551**	0.0698	0.0671	0.0686	0.0532	0.064	0.0612	0.0446	0.0657	0.0646	0.0566	**0.04**	0.0579	0**.0429**

	Worst							SD						
	ACO	PSO	WOA	DMO	ARO	HHO	CDMO8	ACO	PSO	WOA	DMO	ARO	HHO	CDMO8
base_exactly	0.2478	**0.219**	0.2435	0.3175	0.2528	0.2561	0.29	0.0708	0.0289	**0.0214**	0.0491	0.0553	0.0252	0.1858
base_BreastEW	0.0332	0.029	**0.0251**	0.0297	0.0336	0.0301	0.0265	0.0034	0.0038	**0.0014**	0.0040	0.0054	0.0026	0.0034
base_M-of-n3	0.0952	0.0936	0.0775	0.095	0.0788	0.088	**0.03**	0.0218	**0.0105**	0.0116	0.0209	0.0117	0.0111	0.0116
BreastEW	0.0302	**0.0297**	0.0328	0.0299	0.0336	0.0312	0.0354	0.0034	0.0028	**0.0024**	0.0051	0.0048	0.0026	0.0055
KrvskpEW	0.0394	0.04047	0.0368	0.0518	0.0539	0.0445	**0.0203**	0.0033	0.0031	**0.0017**	0.0081	0.0095	0.0024	0.0029
SonarEW	0.1317	0.1034	0.0976	0.1015	0.0985	0.1065	**0.0488**	0.0154	0.0146	**0.0054**	0.0201	0.0206	0.01	0.0130
SpectEW	0.1381	**0.1102**	0.1245	0.1195	0.1283	0.1241	0.1132	0.0089	0.0056	**0.0032**	0.0098	0.0103	0.0044	0.0136
Waveform	0.1599	0.1585	0.1594	**0.1334**	0.2337	0.169	0.1378	**0.0028**	0.0045	0.0031	0.0084	0.0130	0.0038	0.012
CongressEW	0.0421	0.0421	0.0374	0.0349	0.0372	0.0387	**0.0345**	0.0042	0.0042	**0.0024**	0.0048	0.0042	0.0033	0.0061
Ionosphere	0.0971	0.0977	0.0914	0.1149	0.1034	0.1009	**0.1143**	**0.0134**	0.0071	0.0059	0.0138	0.0175	0.0065	0.0128

Significant values are in bold.

Table 4 shows the accuracy of the average runs for the ten CDMO where the number after CDMO refers to the map number in Table 1, for example CDMO1 is Chebyshev map. Results in Table 4 shows that the Singer map which is CDMO8 has higher results in three datasets named (breastEW, SpectEW, Waveform), CDMO1 and CDMO7 have best results in (KrvskpEW) and (Ionosphere), respectively. All maps have same accuracy in two datasets named (base_exactly) and (base_M-of-n3). Table 5 shows the comparison of average fitness value of the ten chaotic maps. The Singer map (CDMO8) achieved best results in 5 out of 10 datasets. Both CDMO4 and CDMO6 achieved same result in base_M-of-n3. Also, CDMO1, CDMO3, CDMO5, CDMO7, CDMO10 have best results in one dataset for each, so CDMO8 has been chosen to be compared with ACO, PSO, WOA, ARO, HHO and DMO algorithms.

**Figure 3:**
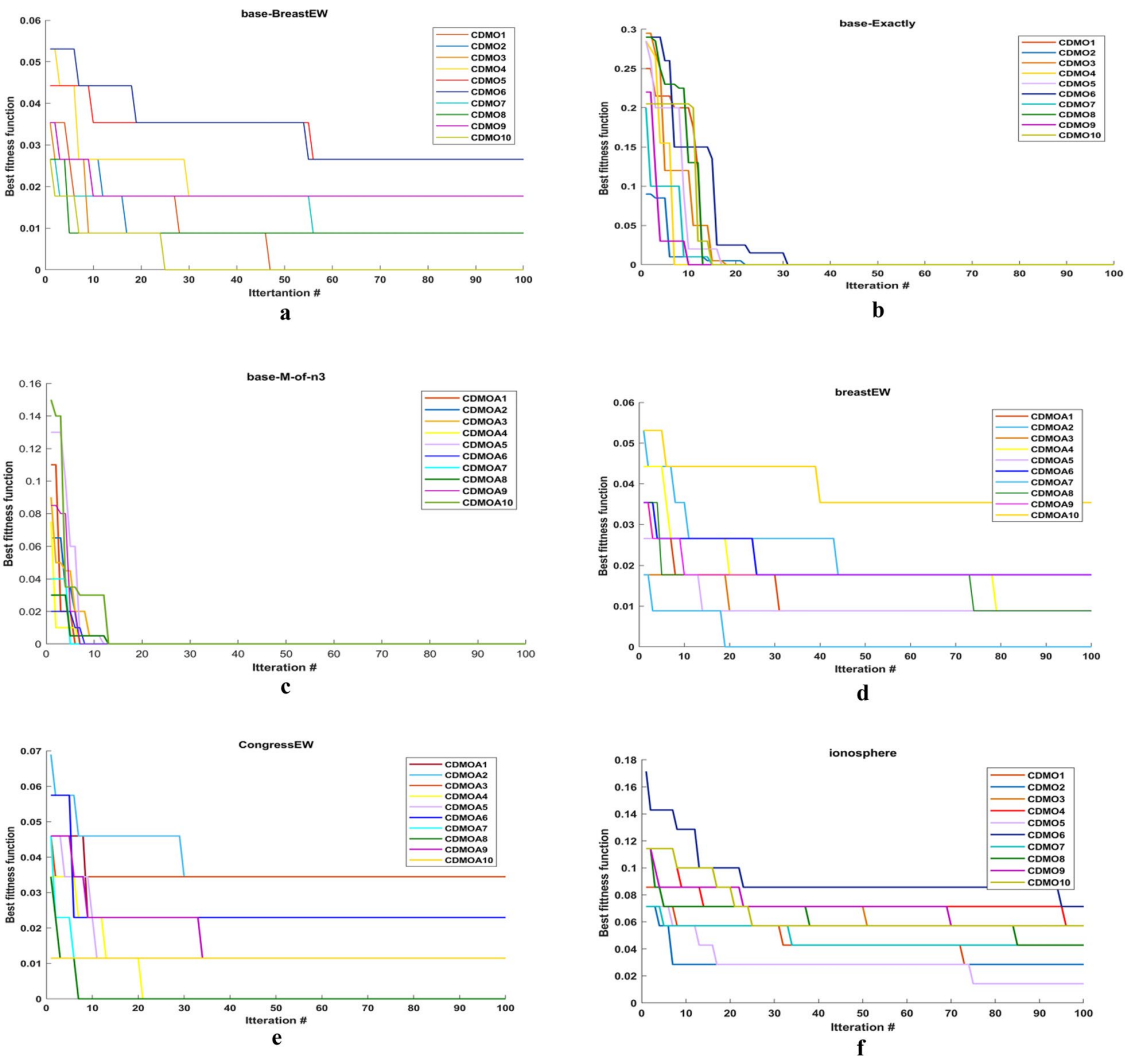

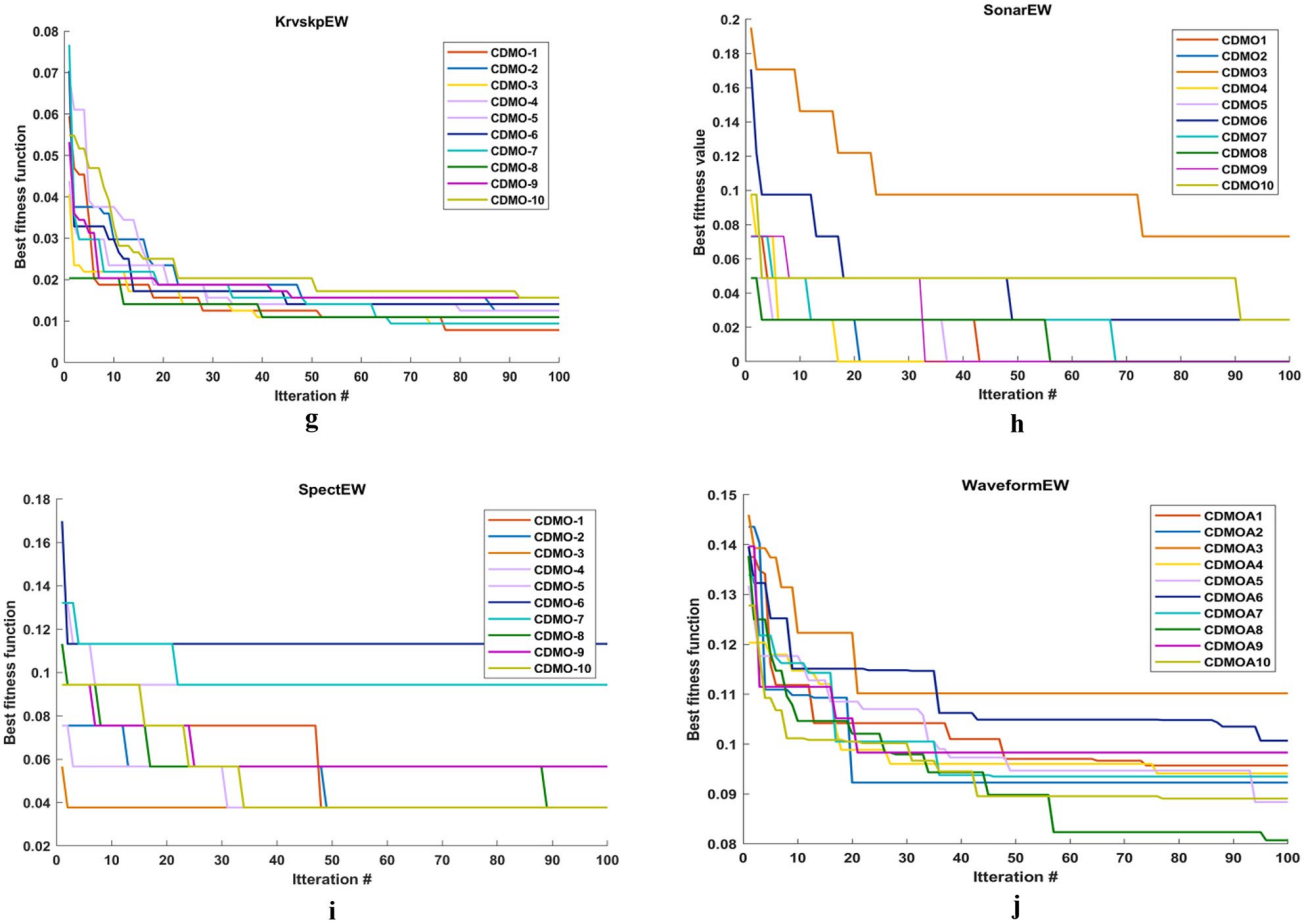
Comparison between ten chaotic maps.

Figure 3 illustrates the convergence curves for the ten chaotic maps. In this figure, the number of iterations is equal to 100. As it can be observed from this figure, almost singer map obtains best result. This is due to that it converges faster than other maps.

### Comparison with other meta-heuristic techniques

In this section, we will compare the performance of the developed method based on Singer map with well-known and most used techniques named PSO, ACO, ARO, HHO and WOA.

From Table 6, the CDMO gives best accuracy in seven datasets (base_BreastEW, SonarEW, SpectEW, Waveform, CongressEW, breastEW and Ionosphere) while DMO gives superior performance in one data set named KrvskpeEW. Moreover, DMO and CDMO give equal performance in 2 datasets (base_M-of-n3 and base_exactly). Based on the results of Precision, CDMO8 has better results in seven datasets. Whereas DMO has better results in one dataset named BreastEW, both CDMO8 and DMO have same results in two datasets. By analysis of the obtained results of the Sensitivity, the CDMO8 has highest results of four datasets, while DMO and PSO have highest results in three datasets and one dataset, respectively. Moreover, both CDMO8 and DMO have same results in two datasets named base_exactly and base_M-of-n3. For specificity results, CDMO8 has highest results in seven datasets while PSO has best results in only one dataset named BreastEW. Besides, both CDMO8 and DMO have same results in two datasets. In addition, F-measure results show that CDMO8 has better results in five datasets while DMO has better result in KrvskpEW dataset and ARO has better result in SpectEW and ionosphere datasets, both CDMO8 and DMO have same results in two datasets.

Table 7 presents the results of fitness metrics which is standard deviation SD, Best, Worst and the Average of fitness function. In the average of fitness function, the CDMO8 achieved best results in 9 out of 10 datasets while ACO has best results in Ionosphere dataset only. In terms of best measure, the CDMO8 has best results in 5 out 10 datasets while the original DMO has best results in 2 out of 10 datasets, ARO has better value in ionosphere and base_M-of-n3 datasets both CDMO8 and DMO have same results in breastEW dataset. Furthermore, for Worst measure, CDMO8 has best results in 5 out of 10 datasets, while PSO has the second rank by 3 out of 10 datasets. WOA and DMO have highest results in one dataset for each. Additionally concerning standard deviation, WOA has the superior results by 7 out of 10 datasets, neither CDMO nor original DMO got best results in standard deviation results.

**Figure 4:**
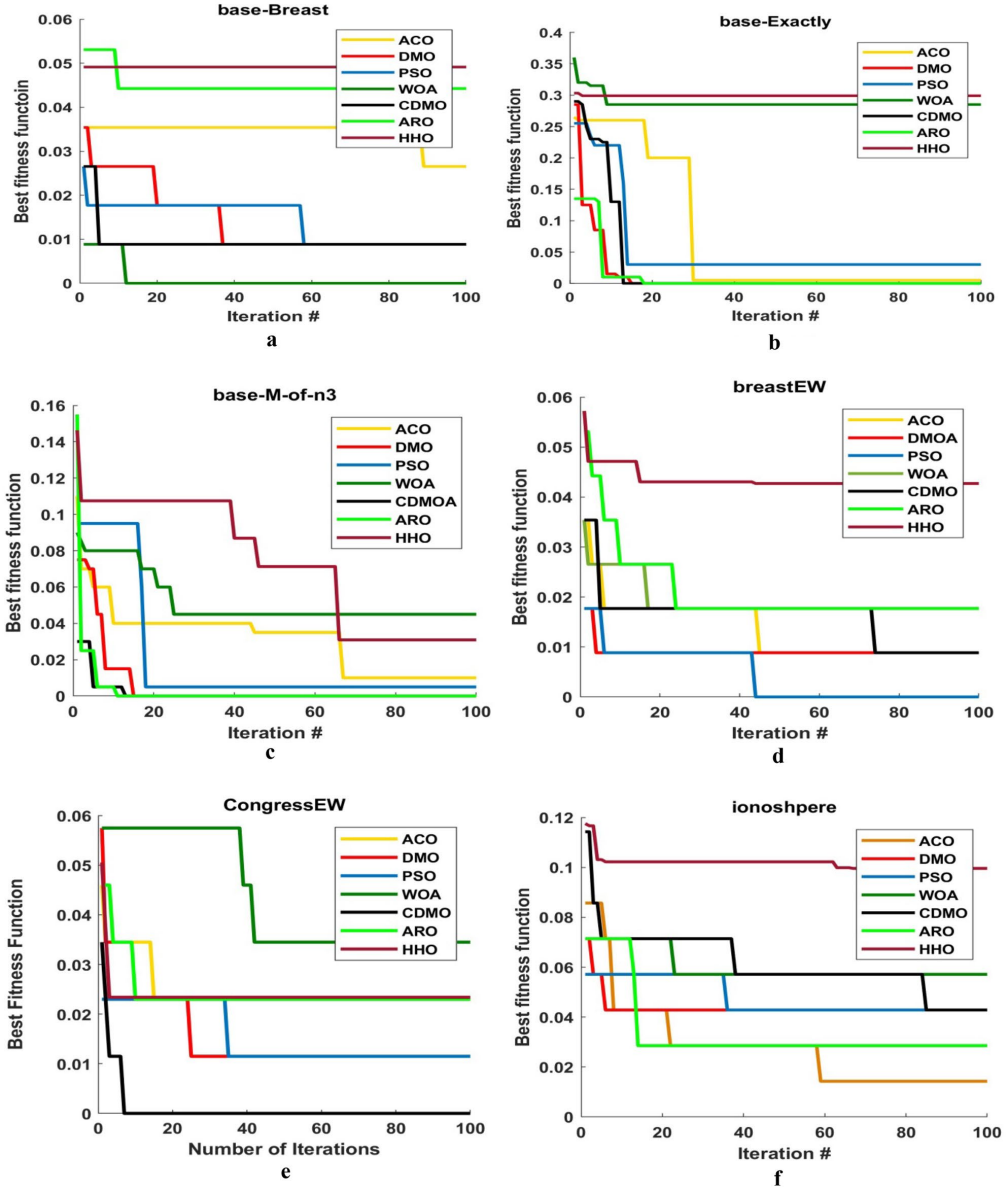

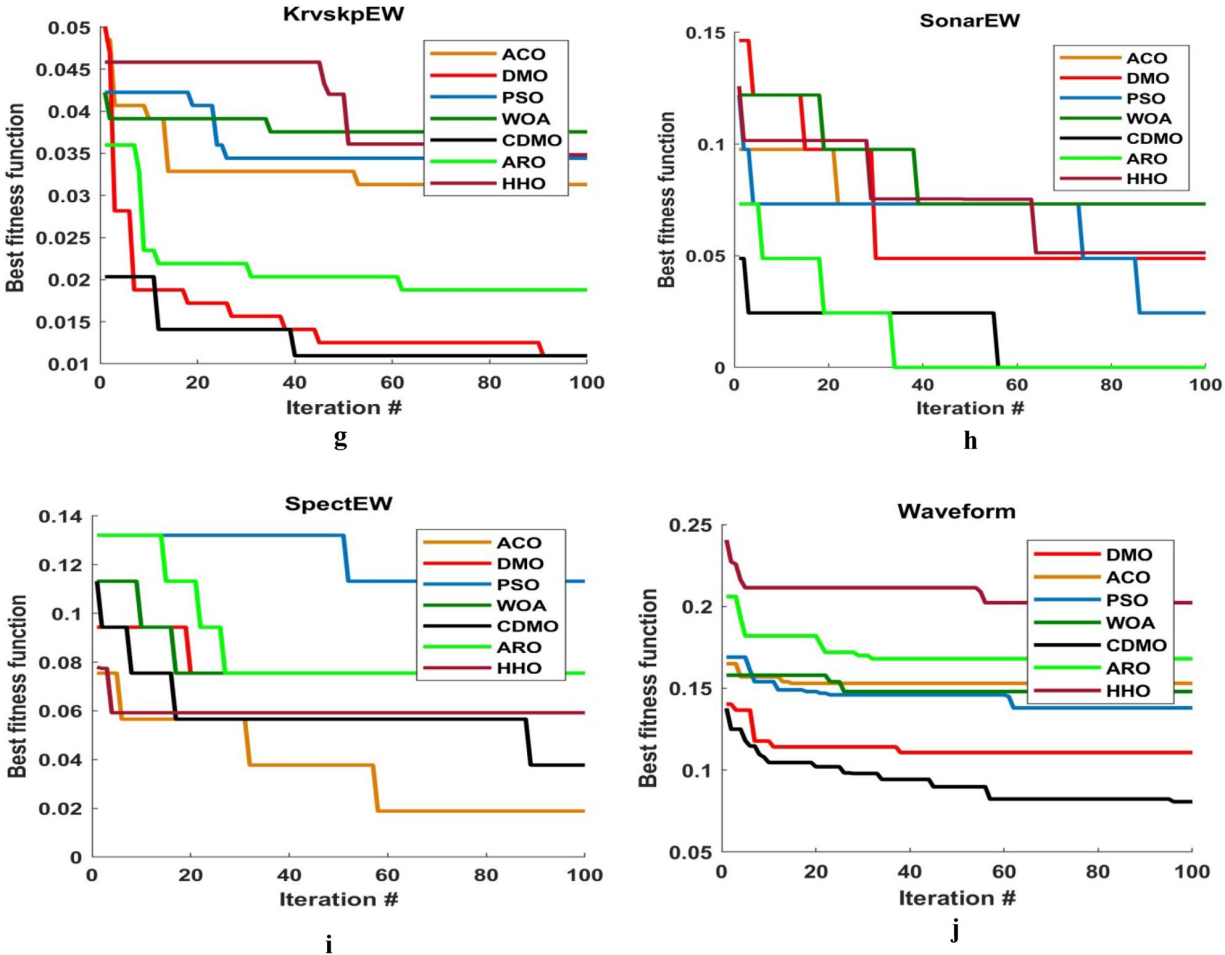
Comparison between best chaotic map and 6 meta-heuristic algorithms.

Figure 4 shows the comparison between CDMO8 and other meta-heuristic algorithms (i.e., PSO, ACO, DMO, ARO, HHO and WOA) in convergence curve. As observed from figure, CDMO8 converges faster in most figures.

Table 8 compares the accuracy of CDMO8 against 6 state-of-the-art methods namely, BGA, RTHS, RSGW, EO, SSAPSO and HSGW. It is clear that our proposed CDMO method stands at the top over these methods. CDMO8 produces higher accuracy in 8 out 10 datasets.

**Table 8:** Comparison of CDMO8 with other 6 state-of-the-art methods based on achieved accuracy (highest classification accuracies are in bold).

Dataset	Accuracy						
	BGA	RTHS	RSGW	EO	SSAPSO	HSGW	CDMO8
base_exactly	**1**	0.997	0.997	0.75	0.967	**1**	**1**
base_BreastEW	0.9743	0.971	0.971	0.9857	0.95	0.986	**0.9911**
base_M-of-n3	**1**	**1**	**1**	0.845	0.978	**1**	**1**
BreastEW	0.9754	098.2	0.982	0.9561	0.9755	0.981	**0.9921**
KrvskpEW	0.985	0.973	0.973	0.8435	0.951	0.973	**0.9890**
SonarEW	0.9904	**1**	0979	0.9048	0.9566	0.964	**1**
SpectEW	0.8955	**0.9815**	0.815	0.8703	0.7913	0.862	0.9722
Waveform	0.7836	0.841	0.757	0.788	0.9620	0.748	**0.9192**
CongressEW	0.9679	**1**	0.961	0.977	0.9686	0.975	**1**
Ionosphere	0.9489	**1**	0.978	0.9571	0.98	0.944	0.9571

### Performance evaluation on CEC’22 benchmark functions

In this section, the performance of the proposed CDMO algorithm in solving optimization problems is tested. To this end, the numerical solving efficiency of CDMO is evaluated by solving twelve functions of CEC’22. The performance of the proposed CDMO on the CEC’22 benchmark function has been determined. Table 9 presents the outcomes for a CEC’2022 test suite for 30 runs performed by the proposed ten chaotic DMO. These benchmark functions consist of four types unimodal, basic, hybrid and composite functions. It is found that CDMO9 achieves the best performance.

**Table 9:** Comparison of simulation outcomes using DMO with 10 chaotic maps for a CEC’2022 test suite for 30 runs.

Fun		CDMO1	CDMO2	CDMO3	CDMO4	CDMO5	CDMO6	CDMO7	CDMO8	CDMO9	CDMO10
*F1*	Mean	37.6703	49.2258	55.2711	24.1597	38.7636	31.7829	41.1619	42.5261	44.4219	49.0182
	STD	− 27.0101	− 25.142	1.4219	− 15.2403	− 19.8947	− 2.752	− 25.7849	− 12.7627	− 18.1099	− 18.616
*F2*	Mean	71.3877	67.4698	72.7695	67.069	70.3485	70.0904	66.9872	67.4974	73.118	69.0384
	STD	28.6156	31.3227	27.4624	31.5527	29.4815	29.5777	31.5268	31.3894	27.7238	30.1431
*F3*	Mean	47.8944	47.8966	47.8976	47.8987	47.8905	47.8964	47.8923	47.8925	47.8869	47.8999
	STD	16.7706	16.77	16.7727	16.7707	16.7623	16.7719	16.7678	16.7747	16.7674	16.7748
*F4*	Mean	31.573	41.3039	18.9391	29.6284	32.9669	38.6676	39.858	22.6606	29.972	38.5803
	STD	− 13.2639	4.7712	− 14.0327	− 4.1446	− 0.44749	4.0023	− 7.8498	− 10.5974	− 8.3744	1.8914
*F5*	Mean	51.20491	46.35887	38.06082	35.40594	35.23488	39.40386	38.17021	58.53465	29.7295	32.53841
	STD	− 8.19181	0.523679	− 2.8823	12.2422	1.414808	− 10.4939	− 0.67047	− 10.3861	− 13.7808	13.33265
*F6*	Mean	31.54436	39.09761	24.29382	23.30663	39.95076	34.20494	43.23234	31.10375	45.60005	29.90376
	STD	1.002241	− 5.60402	− 14.5199	3.995714	− 2.25021	− 7.78239	− 1.25563	4.310759	− 5.67635	− 2.97554
*F7*	Mean	34.85993	49.18917	36.43779	46.29711	25.4561	39.70633	30.87552	43.34505	42.17393	31.99279
	STD	− 3.56281	− 6.69832	− 7.64214	5.231094	− 3.48243	2.88266	− 5.48696	− 1.65038	− 14.488	1.675022
*F8*	Mean	40.14696	43.02149	37.50739	29.97309	45.87442	30.97324	43.92831	49.30015	43.53387	30.326
	STD	4.511035	1.558652	− 13.2439	− 6.44802	− 20.1744	7.831558	11.106	− 5.7076	− 6.19182	− 14.092
*F9*	Mean	45.96478	37.75997	32.08481	44.69134	39.9951	40.51609	42.70853	31.55867	37.03655	42.32642
	STD	− 0.81111	5.822384	− 1.66304	1.246249	− 11.839	− 14.2114	− 11.8797	− 3.09272	− 10.0039	− 2.42989
*F10*	Mean	38.10615	44.71744	39.21914	43.36055	29.22045	39.41734	34.92506	38.68287	43.13838	28.86259
	STD	− 16.5793	− 0.82598	− 0.61679	− 0.3013	12.58064	− 2.10653	− 21.5561	− 7.8547	3.256351	13.59188
*F11*	Mean	44.27529	35.29008	31.00094	23.31097	37.44925	30.37191	32.86412	34.95504	53.42543	41.02269
	STD	3.133682	1.649913	− 6.10969	− 13.5542	3.761989	− 11.6043	− 12.9215	− 5.83714	6.09463	− 5.55082
*F12*	Mean	− 12.6973	0.211765	6.528745	− 3.575	− 4.6557	− 10.972	− 12.2923	− 12.2741	2.576182	6.86794
	STD	37.70109	35.08934	36.39644	39.99058	30.95312	36.44769	36.40298	33.68914	46.62329	41.95957

In order to verify the effectiveness of CDMO9, the results of the proposed CDMO9 are compared, in Table 10, with six novel optimization algorithms namely, Artificial Hummingbird Algorithm (AHA)^44^, African Vultures Optimization Algorithm (AVOA)^45^, Crow Search Algorithm (CSA)^46^, Harris Hawks Optimization (HHO)^38^, Northern Goshawk Optimization (NGO)^47^ and Satin Bowerbird Optimizer (SBO)^48^. Besides, in order to demonstrate the ability of CDMO9 to solve optimization problems, the obtained results are compared with two algorithms recently improved by scholars namely, an adaptive quadratic interpolation and rounding mechanism Sine Cosine Algorithm (ARSCA)^49^ and boosting Archimedes Optimization Algorithm using trigonometric operators (SCAOA)^50^. The experimental results show that the proposed method compares favorably with these methods.

**Table 10:** Comparison of simulation outcomes for a CEC’2022 test suite for 30 runs (highest classification accuracies are in bold).

Fun		AHA	AVOA	CSA	HHO	NGO	SBO	ARSCA	SCAOA	CDMO9
*F1*	Mean	3.00E+02	3.00E+02	6.04E+03	3.00E+02	3.00E+02	3.00E+02	3.00E+02	3.23E+02	**4.421**E+01
	STD	1.34E−11	7.24E−14	2.68E+03	1.36E−01	5.59E−14	2.13E−01	1.96E−02	1.79E+01	− **1.810**E+01
*F2*	Mean	4.07E+02	4.16E+02	6.10E+02	4.22E+02	4.04E+02	4.10E+02	4.05E+02	4.04E+02	**7.311**E+01
	STD	1.76E+01	2.70E+01	9.44E+01	2.91E+01	1.30E+01	2.09E+01	1.31E+01	**2.95E+00**	2.772E+01
*F3*	Mean	6.00E+02	6.04E+02	6.28E+02	6.18E+02	6.00E+02	6.04E+02	6.04E+02	6.01E+02	**4.788**E+01
	STD	9.71E−03	3.85E+00	6.14E+00	1.19E+01	1.94E−01	7.05E+00	3.31E+00	**1.89E**−**01**	1.676E+01
*F4*	Mean	8.23E+02	8.26E+02	8.35E+02	8.29E+02	8.09E+02	8.27E+02	8.20E+02	8.11E+02	**2.997**E+01
	STD	7.64E+00	9.33E+00	9.63E+00	7.39E+00	2.86E+00	9.69E+00	6.29E+00	2.90E+00	− **8.374**E+00
*F5*	Mean	9.22E+02	1.03E+03	1.11E+03	1.38E+03	9.00E+02	1.33E+03	9.10E+02	9.00E+02	**2.972**E+01
	STD	4.29E+01	1.14E+02	8.43E+01	2.07E+02	1.55E+00	2.72E+02	2.05E+01	2.01E−01	− **1.378**E+01
*F6*	Mean	2.05E+03	3.42E+03	3.41E+05	2.99E+03	1.97E+03	2.51E+03	3.53E+03	2.96E+03	**4.560**E+01
	STD	4.79E+02	1.38E+03	1.07E+06	1.44E+03	2.24E+02	9.57E+02	1.94E+03	1.00E+03	− **5.676**E+00
*F7*	Mean	2.01E+03	2.03E+03	2.05E+03	2.03E+03	2.01E+03	2.05E+03	2.02E+03	2.02E+03	**4.2173**E+01
	STD	9.42E+00	1.03E+01	1.48E+01	1.11E+01	6.67E+00	4.54E+01	8.10E+00	6.26E+00	− **1.4488**E+01
*F8*	Mean	2.22E+03	2.22E+03	2.23E+03	2.23E+03	2.22E+03	2.27E+03	2.22E+03	2.22E+03	**4.353**E+01
	STD	6.78E+00	6.53E+00	4.53E+00	9.55E+00	8.82E+00	8.77E+01	6.91E+00	8.02E+00	− **6.1912**E+00
*F9*	Mean	2.53E+03	2.53E+03	2.65E+03	2.55E+03	2.53E+03	2.53E+03	2.53E+03	2.53E+03	**3.7036**E+01
	STD	1.64E−10	9.11E+00	3.06E+01	5.08E+01	4.63E−13	2.68E+01	2.68E+01	7.08E+00	− **1.0003**E+01
*F10*	Mean	2.50E+03	2.50E+03	2.51E+03	2.61E+03	2.53E+03	2.69E+03	2.54E+03	2.51E+03	**4.3138**E+01
	STD	**1.17E**−**01**	1.34E−01	8.11E+00	7.46E+01	4.67E+01	1.97E+02	5.78E+01	3.67E+01	3.256 E+00
*F11*	Mean	2.62E+03	2.64E+03	2.92E+03	2.80E+03	2.64E+03	2.74E+03	2.66E+03	2.61E+03	**5.3425**E+01
	STD	8.07E+01	6.48E+01	8.71E+01	1.33E+02	7.75E+01	1.62E+02	1.31E+02	1.60E+01	**6.094**E+00
*F12*	Mean	2.87E+03	2.88E+03	2.89E+03	2.89E+03	2.86E+03	2.95E+03	2.87E+03	2.86E+03	**2.5761**E+00
	STD	4.97E+00	8.90E+00	1.48E+01	2.61E+01	1.65E+00	5.33E+01	5.98E+00	**1.34E+00**	4.6623E+01

## Conclusion and future work

Chaotic Dwarf Mongoose Optimization Algorithm (CDMO) was proposed which is Dwarf Mongoose algorithm hybridized by chaos. To enhance the performance of the proposed technique, ten chaotic maps were employed where CDMO is used as a wrapper feature selector. The CDMO gives superior performance than the well-known meta-heuristic algorithms, namely PSO, ACO, WOA, ARO, HHO BGA, RTHS, RSGW, EO, SSAPSO, HSGW and DMO. The obtained results proved that the capability of CDMO to select the best feature set gives high classification results. Moreover, the experimental results proved that the adjusted variable using the Singer map significantly enhanced the DMO algorithm in terms of classification performance, and fitness performance. Moreover, our proposed algorithm is tested using the recent optimizers in CEC’22.

In the future work we can extend this work to solve real world problem like medical data. In addition, it would be interested to investigate in hybridization DMO algorithm with another swarm meta-heuristic algorithm.

### Ethics approval

This research contains neither human nor animal studies.

## Data Availability

The datasets used in this study are available in the UC Irvine Machine Learning Repository, “https://archive.ics.uci.edu/: Access Date: 10 May 2023. “
